# Regulation of type I interferon signature by VGLL3 in the fibroblast-like synoviocytes of rheumatoid arthritis patients via targeting the Hippo pathway

**DOI:** 10.1186/s13075-022-02880-0

**Published:** 2022-08-08

**Authors:** Yu Du, Ran Cui, Na Tian, Miao Chen, Xian-Long Zhang, Sheng-Ming Dai

**Affiliations:** 1 Department of Rheumatology and Immunology, Shanghai Jiao Tong University Affiliated Sixth People’s Hospital, 600 Yishan Road, Shanghai, 200233 China; 2 Department of Orthopedic Surgery, Shanghai Jiao Tong University Affiliated Sixth People’s Hospital, Shanghai, China

**Keywords:** Rheumatoid arthritis, Interferon beta, Type I interferon signature, Hippo pathway

## Abstract

**Background:**

The upregulation of interferon (IFN)-stimulated genes induced by type I IFNs (namely type I IFN signature) in rheumatoid arthritis (RA) patients had implications in early diagnosis and prediction of therapy responses. However, factors that modulate the type I IFN signature in RA are largely unknown. In this study, we aim to explore the involvement of VGLL3, a homologue of the vestigial-like gene in Drosophila and a putative regulator of the Hippo pathway, in the modulation of type I IFN signature in the fibroblast-like synoviocytes (FLS) of RA patients.

**Methods:**

FLS were isolated from RA and osteoarthritis (OA) patients. Expression of VGLL3 in the synovial tissues and FLS was analyzed by immunohistochemistry and PCR. RNA sequencing was performed in RA-FLS upon VGLL3 overexpression. The expression of IFN-stimulated genes was examined by PCR and Western blotting.

**Results:**

VGLL3 was upregulated in the RA synovium and RA-FLS compared to OA. Overexpression of VGLL3 promoted the expression of IFN-stimulated genes in RA-FLS. The expression of STAT1 and MX1 was also upregulated in RA synovium compared to OA and was associated with the expression of VGLL3 in RA and OA patients. VGLL3 promoted the IRF3 activation and IFN-β1 expression in RA-FLS. Increased IFN-β1 induced the expression of IFN-stimulated genes in RA-FLS in an autocrine manner. VGLL3 also modulated the expression of the Hippo pathway molecules WWTR1 and AMOTL2, which mediated the regulation of IRF3 activation and IFN-β1 production by VGLL3 in RA-FLS.

**Conclusions:**

VGLL3 drives the IRF3-induced IFN-β1 expression in RA-FLS by inhibiting WWTR1 expression and subsequently promotes the type I IFN signature expression in RA-FLS through autocrine IFN-β1 signaling.

## Background

Rheumatoid arthritis (RA) is an inflammatory autoimmune disease that predominantly affects the diarthrodial joints, characterized by synovitis and destructive erosion of cartilage and bone [1]. The chronic synovitis in RA exhibits distinctive features, including the expansion of the fibroblast-like synoviocytes (FLSs) population, infiltration of leucocytes, and elevated expression of inflammatory mediators such as cytokines and chemokines [2]. Type I interferons (IFNs), predominantly consisting of α and β subtypes, were initially recognized as master inducers of anti-viral responses. Nevertheless, growing evidence revealed their potential role in the pathogenesis of RA [3, 4]. After engagement with the IFN-α receptor (IFNAR), type I IFNs canonically activate the IFN-stimulated gene factor 3 (ISGF3) complex composed of signal transducer and activator of transcription 1 (STAT1), STAT2, and IFN regulatory factor 9 (IRF9). The ISGF3 complex subsequently induces the expression of a broad spectrum of IFN-stimulated genes (ISGs) [5]. ISGs belong to a set of nearly two thousand genes regulated by different IFNs in a subtype-dependent manner [6, 7]. The upregulation of ISGs induced by type I IFNs (namely type I IFN signature) participated in the RA pathogenesis and had implications in early diagnosis and prediction of therapy responses in RA patients [8]. Among the ISGs, cytokines like tumor necrosis factor ligand superfamily member 13B (TNFSF13B; also known as B cell-activating factor, BAFF), and chemokines such as CCL5, CXCL10, and CXCL12, could be continuously secret by FLSs in the inflamed RA synovium. They are at least partly responsible for the recruitment of leucocytes to the synovium and the perpetuation of the local inflammation [9, 10]. Furthermore, STAT1, which is an IFN signal transducer and an ISG, was found to be upregulated and activated in the RA synovial fluid and synovium [11, 12]. A previous study also showed a type I IFN-dependent activation of STAT1 in RA-FLS [13]. However, the type I IFN signature showed heterogeneity in RA, because it was only found in a subgroup of RA patients [14–16]. The type I IFN signature in the peripheral blood has been recognized as a biomarker of preclinical RA [17]. It was also reported that type I IFN signature negatively predicted the clinical response to rituximab in RA patients [18]. Factors that modulate the type I IFN signature in RA are largely unknown, which impedes the application of type I IFN signature during clinical practice.

There is evidence of the crosstalk between type I IFN signaling and the Hippo pathway [19]. The core proteins of the Hippo pathway in vertebrates are Yes-associated protein (YAP) and its paralog WW domain-containing transcription regulator 1 (WWTR1), which is also known as transcriptional coactivator with PDZ-binding motif (TAZ). Upon activation of this pathway, the upstream large tumor suppressor kinase 1/2 (LATS1/2) phosphorylates YAP/WWTR1, which results in their degradation in the proteasome. Once the upstream signaling is inactivated, YAP/ WWTR1 translocates into the nucleus, leading to transcription of genes that contribute to development, tumorigenesis, and homeostasis [20, 21]. YAP/ WWTR1 was recently reported to inhibit the virus-induced IFN-I and ISGs production [22].

Recently, vestigial family member 3 (VGLL3) has been predicted to have possible connections with the Hippo pathway. VGLL3 is a homologue of the vestigial-like gene in Drosophila. It is a putative transcriptional cofactor for TEA domain-containing transcription factors (TEADs) [23], and it has been implicated in adipocyte differentiation, myogenesis, tumor, and autoimmune diseases [24–26]. Hori et al. revealed that VGLL3 could promote cancer cell proliferation by inhibiting YAP/WWTR1 [27]. VGLL3 was also required in the IFN-α-induced BAFF expression and pathogenesis of systemic lupus erythematosus (SLE) [28]. However, the role of VGLL3 in RA remains unknown. We hypothesized that VGLL3 might be involved in the modulation of type I IFN signature in RA.

Here, we first revealed the upregulation of VGLL3 in RA synovium and further demonstrated that VGLL3 drove the IRF3-induced IFN-β secretion in RA-FLS by inhibiting WWTR1 expression and subsequently promoted type I IFN signature expression through autocrine IFN-β signaling.

## Methods

### Collection of synovial tissues and culture of human FLS

Synovial tissues were collected from ten RA patients (all females, mean age 60.2 ± 11.8 years) and five osteoarthritis (OA) patients (all females, mean age 64.2 ± 7.1 years) diagnosed according to the1987 ACR classification criteria for RA [29] or 1986 revised ACR classification criteria for knee OA [30] during total knee arthroplasty in the department of orthopedic surgery of Shanghai Jiao Tong University Affiliated Sixth People’s Hospital. All the patients were not receiving any antirheumatic drug therapy at the time of surgery. Informed consent was obtained from all patients before sample collection. This study complied with the Declaration of Helsinki (1964), and the research was approved by the Ethic Committee of Shanghai Jiao Tong University Affiliated Sixth People’s Hospital. FLS were isolated from synovial tissues by digesting with 1 mg/mL type II collagenase (Merck Millipore, MA, USA) as previously described [31]. Cells were collected and cultured in Dulbecco’s Modified Eagle Medium (DMEM, Hyclone, UT, USA) containing 10% fetal bovine serum (FBS, Gibco, NY, USA). FLS at passages 3–8 were used in the following experiments.

### Immunohistochemistry and immunofluorescence staining

Synovial tissues were fixed in paraformaldehyde (PFA) and embedded in paraffin. For immunohistochemistry, paraffin-embedded sections were blocked with 10% donkey serum (Jackson ImmunoResearch Labs, PA, USA) and incubated with rabbit anti-VGLL3 antibody (1:500, HPA054983, Merck Millipore, MA, USA), rabbit anti-MX1 antibody (1:100, 13750-1-AP, Proteintech, Wuhan, China), rabbit anti-STAT1 antibody (1:250, ab109320, Abcam, Cambridge, UK), and rabbit IgG (isotype control, 12-370, Merck Millipore, MA, USA) at 4 °C overnight. After washing with phosphate-buffered saline (PBS) three times, sections were incubated with horseradish peroxidase (HRP)-conjugated donkey anti-rabbit antibody (1:1000, Jackson ImmunoResearch Labs, PA, USA) and further stained with diaminobenzidine (DAB, Beyotime Biotechnology, Shanghai, China). For immunofluorescence staining, cultured FLS were fixed in PFA and permeabilized with 0.5% Triton X-100. After blocking with 10% donkey serum, cells were incubated with rabbit anti-VGLL3 antibody (1:500, ab83555, Abcam, Cambridge, UK), rabbit anti-STAT1 antibody ((1:250, ab109320, Abcam, Cambridge, UK), rabbit anti-phospho-STAT1 antibody (1:1000, 7649, Cell Signaling Technology, MA, USA), and rabbit anti-IRF3 antibody (1:100, ab76493, Abcam, Cambridge, UK) at 4°C overnight. The next day, cells were washed with PBS and incubated with Alexa Fluor 555-conjugated donkey anti-rabbit antibody (1:500, ab150130, Abcam, Cambridge, UK) and mounted with Antifade Mounting Medium with DAPI (4’,6-diamidino-2-phenylindole) (Beyotime Biotechnology, Shanghai, China). Images were captured with a microscope (Olympus, Tokyo, Japan) (magnification ×200) with an equal time of exposure.

### siRNA transfection and lentivirus transduction

VGLL3 siRNA or WWTR1 siRNA were transfected into RA-FLS to knock down the expression of these genes. Scramble siRNA was used as the negative control. All the siRNA were synthesized by Ribobio (Guangzhou, China). The target sequences are as follows: scramble siRNA, TTCTCCGAACGTGTCACGT; VGLL3 siRNA, GGTCAGTAGTGGATGAACA; WWTR1 siRNA, CGATGAATCAGCCTCTGAA. siRNA was transfected into FLS using Lipofectamine 2000 (Thermo Fisher Scientific, MA, USA) transfection reagent following the manufacturer’s instructions. For VGLL3 overexpression, full-length VGLL3 was cloned into lentiviral vectors (pHBLV-CMV-MCS-3FLAG-EF1-ZsGreen-T2A-PURO) via standard molecular biology techniques. Lentivirus was produced by co-transfecting 293T cells with VGLL3 lentiviral vectors or control vectors together with packaging plasmids. The lentivirus solution was concentrated by ultracentrifugation. The titers of control and overexpression lentivirus solutions were determined by infecting 293T cells. Briefly, 293T cells were seeded in a 96-well plate at a density of 10^4^ cells/well and added with gradient diluted virus solutions in different wells. The titers of viruses were calculated after 72 h with 10%-50% GFP-positive wells using the following formula:

$$\mathrm{Titers}\;\left(\mathrm{TU}/\mathrm{mL}\right)=\mathrm{cell}\;\mathrm{counts}\;\mathrm{per}\;\mathrm{well}\times\mathrm{percentage}\;\mathrm{of}\;\mathrm{GFP}-\mathrm{positive}\;\mathrm{cells}\times\mathrm{MOI}\;(1)\times\mathrm{virus}\;\mathrm{dilution}\;\mathrm{ratio}\times10^3\;\mathrm{TU}/\mathrm{mL}$$

FLS were infected with control or VGLL3-overexpressing lentiviruses at an MOI of 300 with 5μg/ml polybrene.

### RNA extraction, reverse transcription PCR (RT-PCR), and real-time quantitative PCR (qPCR)

Total RNA was extracted by TRIzol reagent (Thermo Fisher Scientific, MA, USA) according to the manufacturer’s instructions. cDNA was generated using the ReverTra Ace qPCR RT Master Mix with gDNA Remover kit (Toyobo, Osaka, Japan). RT-PCR was conducted by 2×Hieff^TM^ PCR Master Mix (Yeasen, Shanghai, China) followed by agarose gel electrophoresis. qPCR was performed using SYBR Green Realtime PCR Master Mix kit (Toyobo, Osaka, Japan). The primer sequences are listed in Table 1.

**Table 1:** Primer sequences for PCR

Gene symbol	Forward primer (5′-3′)	Reverse primer (5′-3′)
β-actin	GGACCTGACTGACTACCTCAT	CGTAGCACAGCTTCTCCTTAAT
GAPDH	GAATGGGCAGCCGTTAGGAA	AAAAGCATCACCCGGAGGAG
VGLL3	GGAGACATTGGGTCAGTAGTGG	GGGTTAGCCCCATCTTGCTT
STAT1	ATCAGGCTCAGTCGGGGAATA	TGGTCTCGTGTTCTCTGTTCT
TNFSF13B	GGGAGCAGTCACGCCTTAC	GATCGGACAGAGGGGCTTT
MX1	GGTGGTCCCCAGTAATGTGG	CGTCAAGATTCCGATGGTCCT
IRF7	CCCACGCTATACCATCTACCT	GATGTCGTCATAGAGGCTGTTG
OAS1	TGTCCAAGGTGGTAAAGGGTG	CCGGCGATTTAACTGATCCTG
TLR3	CAAACACAAGCATTCGGAATCTG	AAGGAATCGTTACCAACCACATT
CCL5	GCTGCTTTGCCTACATTGCC	TCGGGTGACAAAGACGACTG
IFN-α1	CTTGTGCCTGGGAGGTTGTC	TAGCAGGGGTGAGAGTCTTTG
IFN-β1	GCTTGGATTCCTACAAAGAAGCA	ATAGATGGTCAATGCGGCGTC

### RNA sequencing and data analysis

The purity of extracted RNA was examined by the kaiaoK5500®Spectrophotometer (Kaiao, Beijing, China). RNA integrity and concentration were evaluated using the RNA Nano 6000 Assay Kit of the Bioanalyzer 2100 system (Agilent Technologies, CA, USA). Sequencing libraries were generated using NEBNext® Ultra™ RNA Library Prep Kit for Illumina® (#E7530L, NEB, USA) with 2 μg of total RNA per sample according to the manufacturer’s instructions. The clustering of the index-coded samples was performed on a cBot cluster generation system using HiSeq PE Cluster Kit v4-cBot-HS (Illumina, CA, USA) following the manufacturer’s recommendations. After cluster generation, the libraries were sequenced on an Illumina platform and 150 bp paired-end reads were generated. Reads Count for each gene in each sample was counted by HISAT v2.0.5, and FPKM (fragments per kilobase per million mapped reads) was then calculated to estimate the expression level of genes in each sample. DEGseq v1.18.0 was used for differential gene expression analysis between two samples with nonbiological replicates. Genes with log2 fold change (log_2_(FC)) ≥ 1 and *q* value (false discovery rate or FDR) ≤ 0.05 were identified as differentially expressed genes (DEGs). Heatmaps for the selected DEGs were mapped by R package “pheatmap” in R studio. The expression levels of the DEGs were shown as log_2_(FC). Gene set enrichment analysis (GSEA) for DEGs was performed using the RNA-seq data through WebGestalt as indicated [32], and the top 10 enriched gene sets were demonstrated.

### Western blotting

Cells were lysed by RIPA buffer (Beyotime Biotechnology, Shanghai, China) on ice. Protein concentration was determined using a BCA protein assay kit (Beyotime Biotechnology, Shanghai, China). Denatured proteins were separated by 10% sodium dodecyl sulfate-polyacrylamide gel electrophoresis (SDS–PAGE) and electrotransferred onto polyvinylidene fluoride (PVDF) membranes (Merck Millipore, MA, USA). Membranes were blocked by 10% bovine serum albumin (BSA) and incubated at 4°C overnight with rabbit anti-VGLL3 antibody (1:500, ab83555, Abcam, Cambridge, UK), rabbit anti-STAT1 antibody (1:10000, ab109320, Abcam, Cambridge, UK), rabbit anti-MX1 antibody (1:1000, 13750-1-AP, Proteintech, Wuhan, China), rabbit anti-IRF3 (phosphor S386) antibody (1:1000, ab76493, Abcam, Cambridge, UK), rabbit anti-IRF3 antibody (1:1000, ab68481, Abcam, Cambridge, UK), rabbit anti-YAP/WWTR1 antibody (1:1000, 8418, Cell Signaling Technology, MA, USA), rabbit anti-AMOTL2 antibody (1:500, 23351-1-AP, Proteintech, Wuhan, China), mouse anti-GAPDH antibody (1:5000, T0004, Affinity, OH, USA), and mouse anti-β-Tubulin (1:5000, T0023, Affinity, OH, USA). Next, membranes were washed with Tris-buffered saline (TBS) containing 0.1% Tween 20 and incubated with HRP-conjugated goat anti-rabbit antibody (1:5000, S0001, Affinity, OH, USA) or HRP-conjugated goat anti-mouse antibody (1:5000, S0002, Affinity, OH, USA). Protein bands were detected with ECL substrate (New Cell & molecular Biotech, Suzhou, China) and analyzed using ImageJ software.

### Statistical analysis

All the experiments were conducted at least three times independently. Data were analyzed by IBM SPSS Statistics 22.0 and plotted using GraphPad Prism 8 software. Data were presented as mean ± SD. Student’s *t*-test was used for statistical comparison between two groups. One-way analysis of variance (ANOVA) followed by Dunnett’s T3 post hoc test was adopted for multiple comparisons. *p*-values < 0.05 were considered statistically significant.

## Results

### Upregulated expression of VGLL3 in RA-FLS

**Fig. 1:**
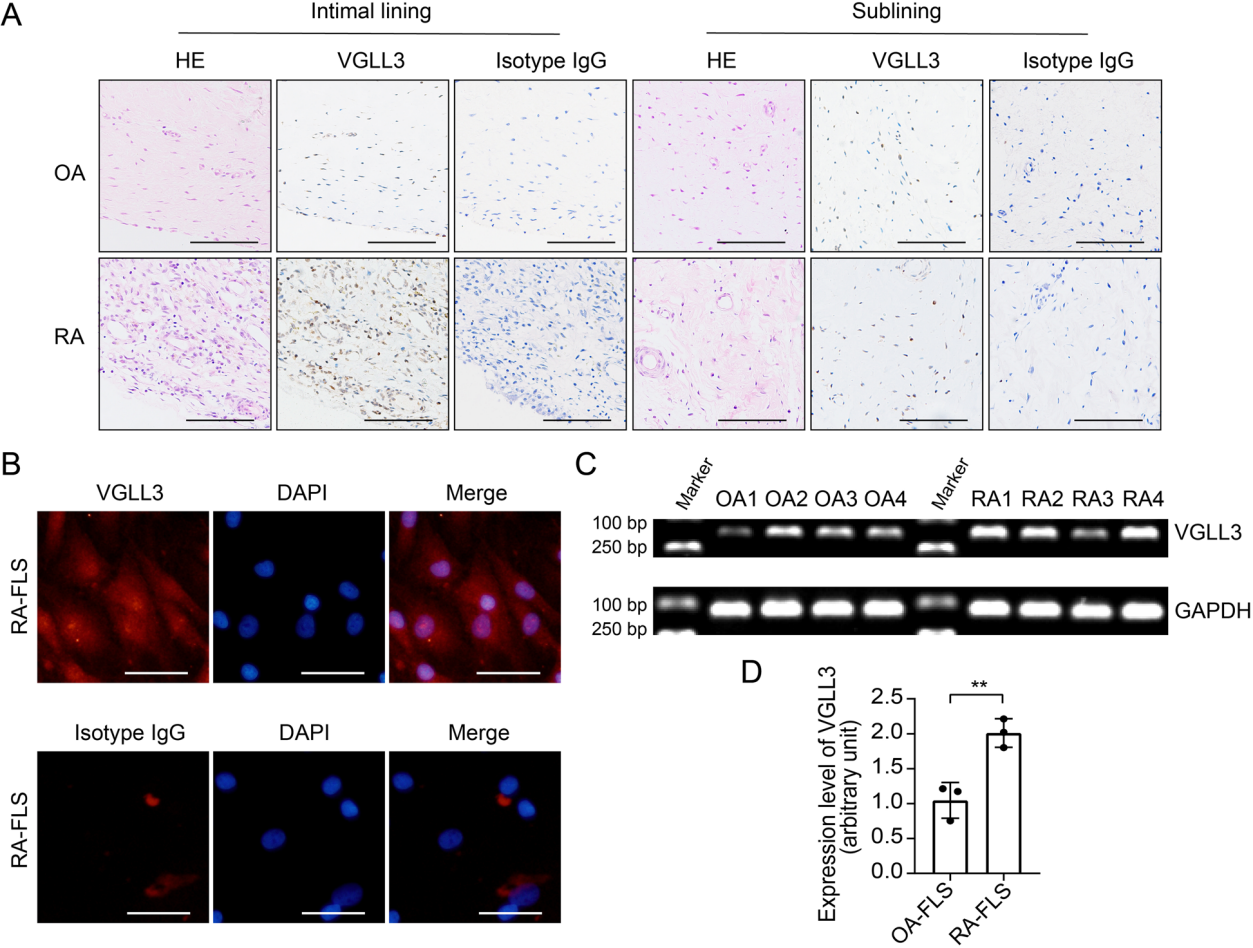
Expression of VGLL3 in the synovial tissues of rheumatoid arthritis (RA) and osteoarthritis (OA) patients. **a** Representative images of the expression of VGLL3 examined by immunohistochemistry staining using anti-VGLL3 antibodies and rabbit IgG (isotype) in RA and OA synovial tissues. Hematoxylin and eosin (HE) staining was also performed to evaluate the pathological changes. *N* = 5. Scale bar, 500 μm. **b** Representative images of the expression of VGLL3 in the fibroblast-like synoviocytes (FLS) in RA examined by immunofluorescence staining. Nuclei were stained with DAPI. *N* = 3. Scale bar, 50 μm. **c** The mRNA expression of VGLL3 in RA-FLS and OA-FLS was detected by reverse transcription PCR (RT-PCR). GAPDH was used as the housekeeping gene. *N* = 4. **d** The mRNA expression levels of VGLL3 in RA-FLS and OA-FLS were detected by real-time quantitative PCR (qPCR). *N* = 3, ** *p* < 0.01. Data were shown as mean ± standard deviation (SD)

Immunohistochemistry staining was performed to examine the expression of VGLL3 in RA and OA synovium using anti-VGLL3 antibodies and rabbit IgG (isotype). Hematoxylin and eosin (HE) staining was conducted to evaluate the pathological changes. There were significant hyperplasia and leukocyte infiltration in the RA intimal lining layer comparing to OA, and VGLL3 expression was substantially upregulated in RA intimal lining layer (Fig. 1A). However, there was no significant difference in VGLL3 expression in the sublining layer between RA and OA (Fig 1A). Then, the FLS were isolated from RA synovium and subjected to immunofluorescence staining. The results showed that VGLL3 was expressed in both the cytoplasm and nucleus of RA-FLS (Fig. 1B). RT-PCR and qPCR analysis of RA-FLS and OA-FLS at passage 3 also indicated that the mRNA expression level of VGLL3 in RA-FLS was increased approximately by 100% compared with OA-FLS (Fig. 1C, D).

### Regulation of type I IFN signature by VGLL3 in RA-FLS

**Fig. 2:**
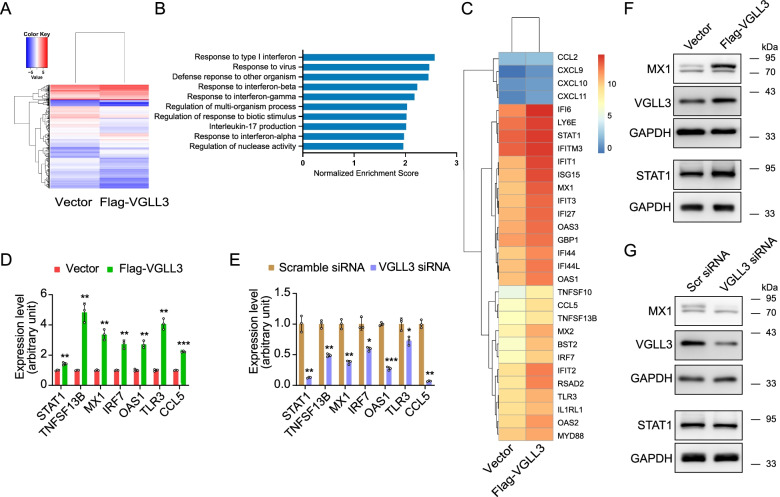
VGLL3-regulated genes in RA-FLS. **a** RNA sequencing (RNA-seq) of RA-FLS transfected with Flag-VGLL3 expressing lentivirus or the vector. The expression levels of the VGLL3-regulated genes were shown as log_2_(FC) in the heatmap. *N* = 1. **b** Gene set enrichment analysis (GSEA) was performed using the RNA-seq data and the top 10 enriched gene sets were demonstrated. **c** The expression levels of the IFN-stimulated genes after VGLL3 overexpression were shown as log_2_(FC) in the heatmap. **d**, **e** The mRNA expression levels of STAT1, TNFSF13B, MX1, IRF7, OAS1, TLR3, and CCL5 were examined by qPCR upon VGLL3 overexpression (**d**) or VGLL3 knockdown (**e**). *N* = 3. **f**, **g** The protein expression levels of VGLL3, STAT1, and MX1 were detected by Western blotting upon VGLL3 overexpression (**f**) or knockdown (**g**). GAPDH was used as the housekeeping gene. Representative images of at least three independent experiments were shown in the Western blotting experiments. * *p* < 0.05, ** *p* < 0.01, *** *p* < 0.001. Scr siRNA: scrambled siRNA. Data were shown as mean ±SD

To address the contribution of VGLL3 to ISGs expression in RA-FLS, VGLL3 was overexpressed in RA-FLS via a lentiviral vector. After 4 days of transfection, Flag-VGLL3-overexpressed RA-FLS and vector-transfected RA-FLS were subjected to RNA sequencing (*n* = 1). The expression levels of all the DEGs were shown as log2(FC) in the heatmap. The RNA expression levels of 1047 genes in total were altered by VGLL3 overexpression, including 357 genes upregulated and 690 genes downregulated (Fig. 2A). Subsequent GSEA revealed that response to type I IFN was the most altered biological process in RA-FLS overexpressing VGLL3 (normalized enrichment score, 2.57, Fig. 2B). Differential gene expression analysis showed that many ISGs were upregulated by VGLL3 overexpression, especially the type I ISGs (Fig. 2C). Real-time PCR verified the upregulation of ISGs (e.g., MX1, OAS1, STAT1, TNFSF13B, IRF7, TLR3, and CCL5) by VGLL3 (Fig. 2D). The protein expression levels of STAT1 and MX1 were also increased by VGLL3 overexpression (Fig. 2F). To exclude the influence on ISGs expression by virus infection, VGLL3 was further silenced by transfection of synthetic siRNA in RA-FLS. The mRNA expression of multiple ISGs and protein expression of STAT1 and MX1 were significantly inhibited after VGLL3 knockdown (Fig. 2E, G).

### The expression levels of STAT1 and MX1 were correlated with VGLL3 in RA synovium

**Fig. 3:**
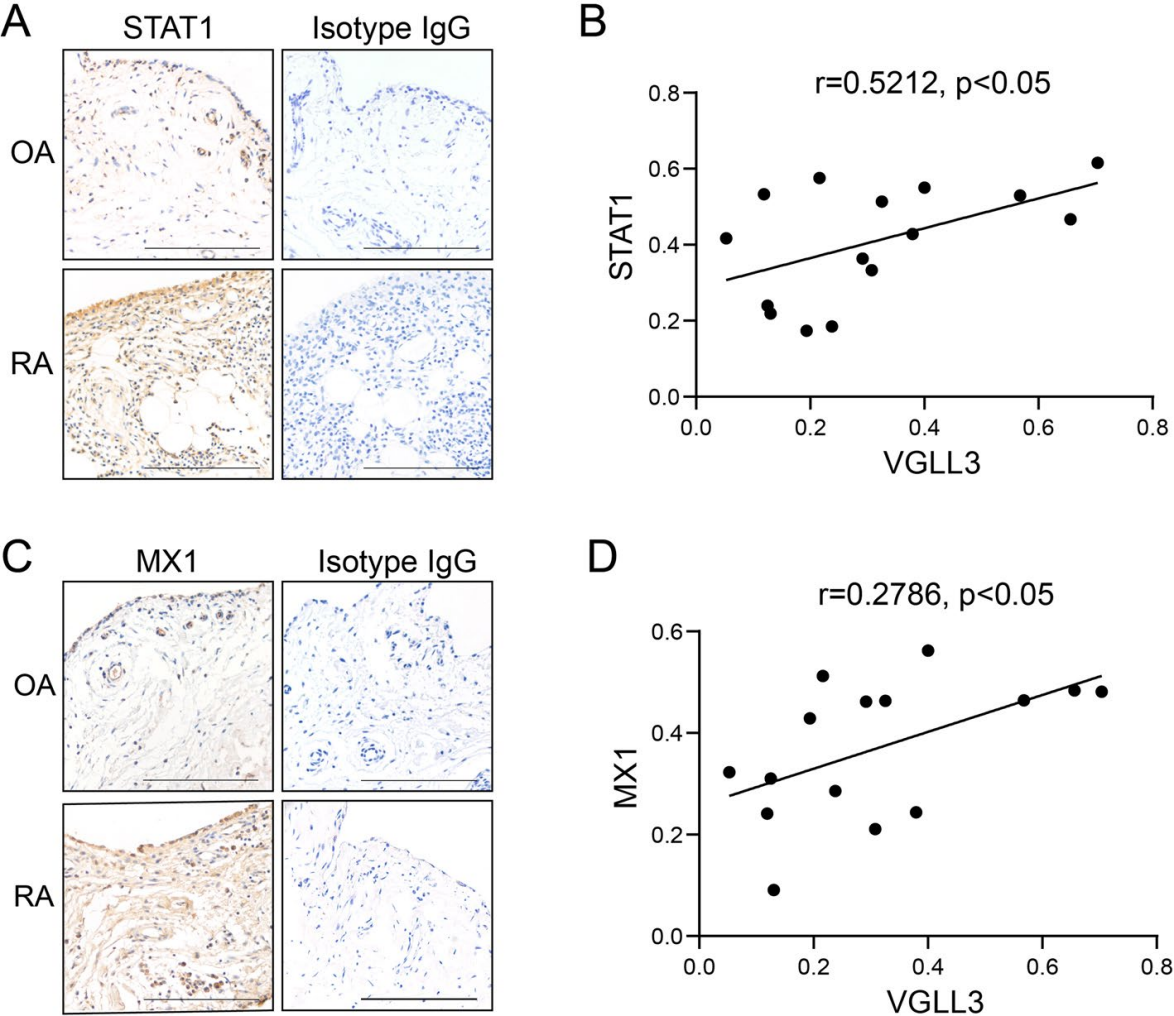
The expression levels of STAT1 and MX1 and their association with VGLL3 expression levels in the synovium of RA and OA patients. **a**, **c** Representative images of the expression of STAT1 (**a**) and MX1 (**c**) examined by immunohistochemistry staining using anti-STAT1 antibodies, anti-MX1 antibodies, and rabbit IgG (isotype) in RA (*n* = 10) and OA (*n* = 5) synovial tissues. Scale bar, 500 μm. **b** The association between the expression levels of STAT1 and VGLL3 in the synovium of ten RA patients and five OA patients. **d** The association between the expression levels of MX1 and VGLL3 in the synovium of ten RA patients and five OA patients

Next, the expression of STAT1 and MX1 in the OA and RA synovium was examined via IHC staining using anti-STAT1 antibody and anti-MX1 antibody. Isotype IgG was used as the negative control. Compared to OA, STAT1 and MX1 were substantially upregulated in the RA synovium (Fig. 3A, C). In both OA and RA patients, the percentage of VGLL3-positive cells had moderate correlations with that of STAT1-positive cells (*r* = 0.5212, *p* < 0.05, Fig. 3B) and was weakly correlated with the proportion of MX1-positive cells (*r* = 0.2786, *p* < 0.05, Fig. 3D). These data indicated that VGLL3 could determine the expression of STAT1 and MX1 in RA synovium to a certain extent.

### VGLL3 facilitating the IRF3-mediated IFN-β production and autocrine signaling in RA-FLS

**Fig. 4:**
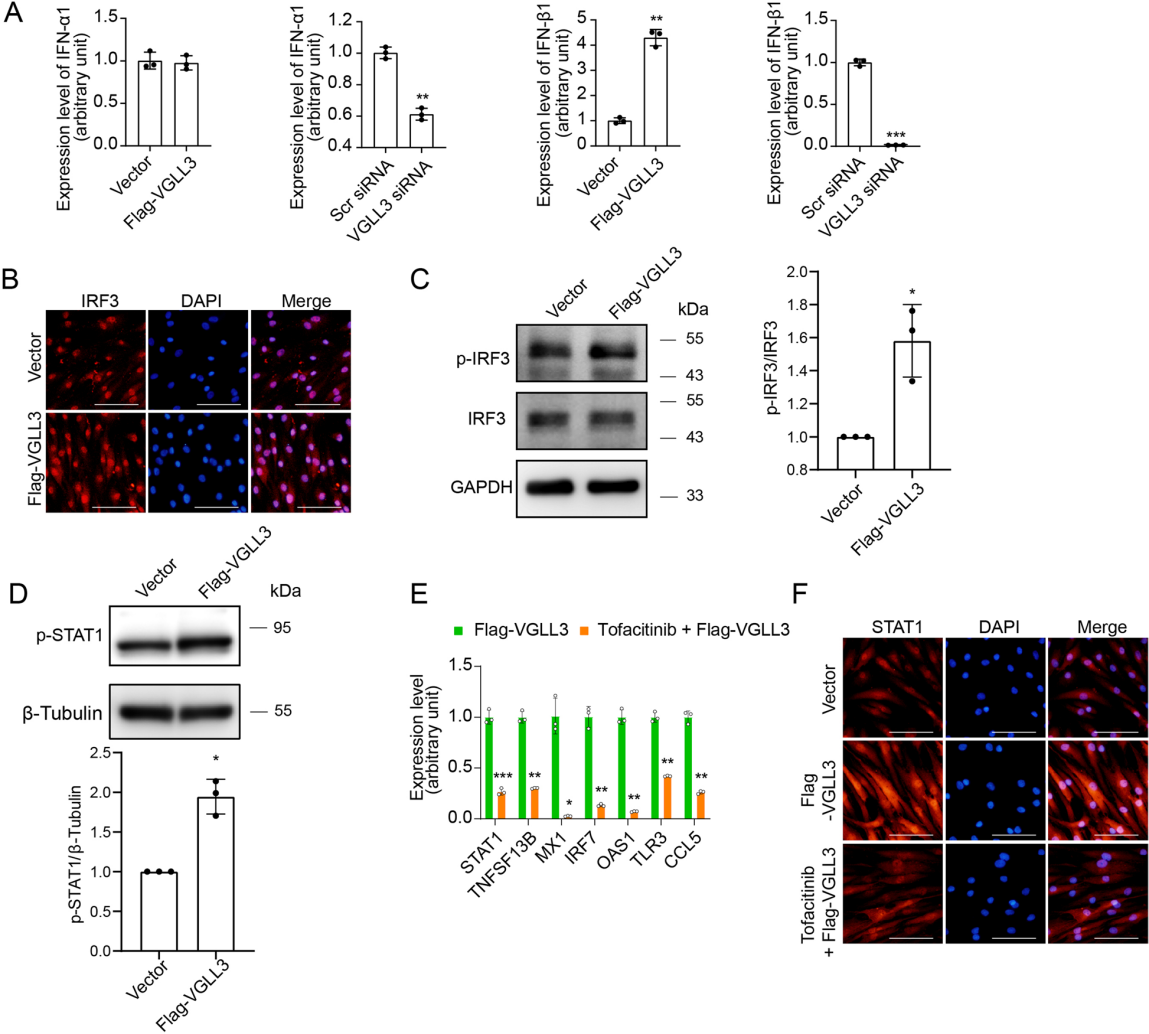
Activation of IRF3 and augmentation of IFN-β1 expression by VGLL3 in RA-FLS. **a** The mRNA expression levels of IFN-α1 and IFN-β1 were detected by qPCR after VGLL3 overexpression or knockdown in RA-FLS. **b** Representative images of the expression of IRF3 in RA-FLS examined by immunofluorescence staining. Nuclei were stained with DAPI. **c** The protein expression levels of p-IRF3 and total IRF3 in RA-FLS were detected by Western blotting upon VGLL3 overexpression. GAPDH was used as the housekeeping gene. **d** The protein expression levels of p-STAT1 in RA-FLS were detected by Western blotting upon VGLL3 overexpression. β-Tubulin was used as the housekeeping gene. **e** Gene expression levels of STAT1, TNFSF13B, MX1, IRF7, OAS1, TLR3, and CCL5 were detected in RA-FLS transfected with Flag-VGLL3-expressing lentivirus with/without the treatment of Janus kinase inhibitor tofacitinib (250 nM). **f** Representative images of the expression of STAT1 in RA-FLS transfected with Flag-VGLL3-expressing lentivirus with/without the treatment of tofacitinib (250 nM) examined by immunofluorescence staining. Nuclei were stained with DAPI. Scale bar, 100 μm. *N* = 3. * *p* < 0.05, ** *p* < 0.01, *** *p* < 0.001

The mechanism that mediates the VGLL3-enhanced ISGs expression in RA-FLS remains unelucidated. Given that response to type I IFN was the predominantly modified biological process by VGLL3 overexpression, it was then explored whether VGLL3 changed the expression level of type I IFN. VGLL3 was overexpressed via a lentiviral vector or silenced by transfected with VGLL3 siRNA in RA-FLS. The mRNA expression of IFN-α1 (gene name, IFNA1) and IFN-β1 (gene name, IFNB1) in RA-FLS after overexpression or knockdown of VGLL3 was examined via qPCR. VGLL3 overexpression significantly increased the expression of IFN-β1 in RA-FLS, but the expression of IFN-α1 was unaffected. Correspondingly, VGLL3 knockdown considerably decreased the IFN-β1 expression level, and the expression of IFN-α1 was only moderately inhibited (Fig. 4A). These results indicated that VGLL3 might enhance the expression of ISGs in RA-FLS by facilitating IFN-β1 production. IRF3 is a master transcription factor for IFN-β expression. The nuclear translocation and phosphorylation of IRF3 were further examined by immunofluorescence staining after overexpression of VGLL3. Nuclei were stained with DAPI. The results showed that overexpression of VGLL3 markedly increased the protein level of IRF3 in the nuclei of RA-FLS (Fig. 4B), and the phosphorylation of IRF3 in RA-FLS was also enhanced by overexpression of VGLL3 as shown by Western blotting (Fig. 4C). STAT1 could be phosphorylated and activated upon IFN-β stimulation and translocated into the nuclear to activate the expression of ISGs. It was found that overexpression of VGLL3 promoted the phosphorylation of STAT1 (Fig. 4D), indicating the activation of STAT1. To verify that the elevated level of IFN-β1 after overexpression of VGLL3 was accountable for the increased expression of ISGs, a Janus kinase (JAK) inhibitor tofacitinib was used to block the intracellular signaling transduction of the IFN-β1 receptor IFNAR. RA-FLS were transfected with Flag-VGLL3-expressing lentivirus with/without the treatment of JAK inhibitor tofacitinib (250 nM). The results demonstrated that VGLL3 overexpression-augmented mRNA expression levels of ISGs (STAT1, BAFF, MX1, IRF7, OAS1, TLR3, and CCL5) were suppressed by tofacitinib (250 nM) (Fig. 4E). And immunofluorescence staining showed that tofacitinib (250 nM) significantly inhibited the expression of STAT1 induced by VGLL3 overexpression (Fig. 4F). The above findings indicated that VGLL3 facilitated the IRF3-mediated IFN-β1 production in RA-FLS, and IFN-β1 might stimulate the expression of ISGs in an autocrine manner.

### VGLL3 exacerbated IFN-β1 production by inhibiting Hippo pathway

**Fig. 5:**
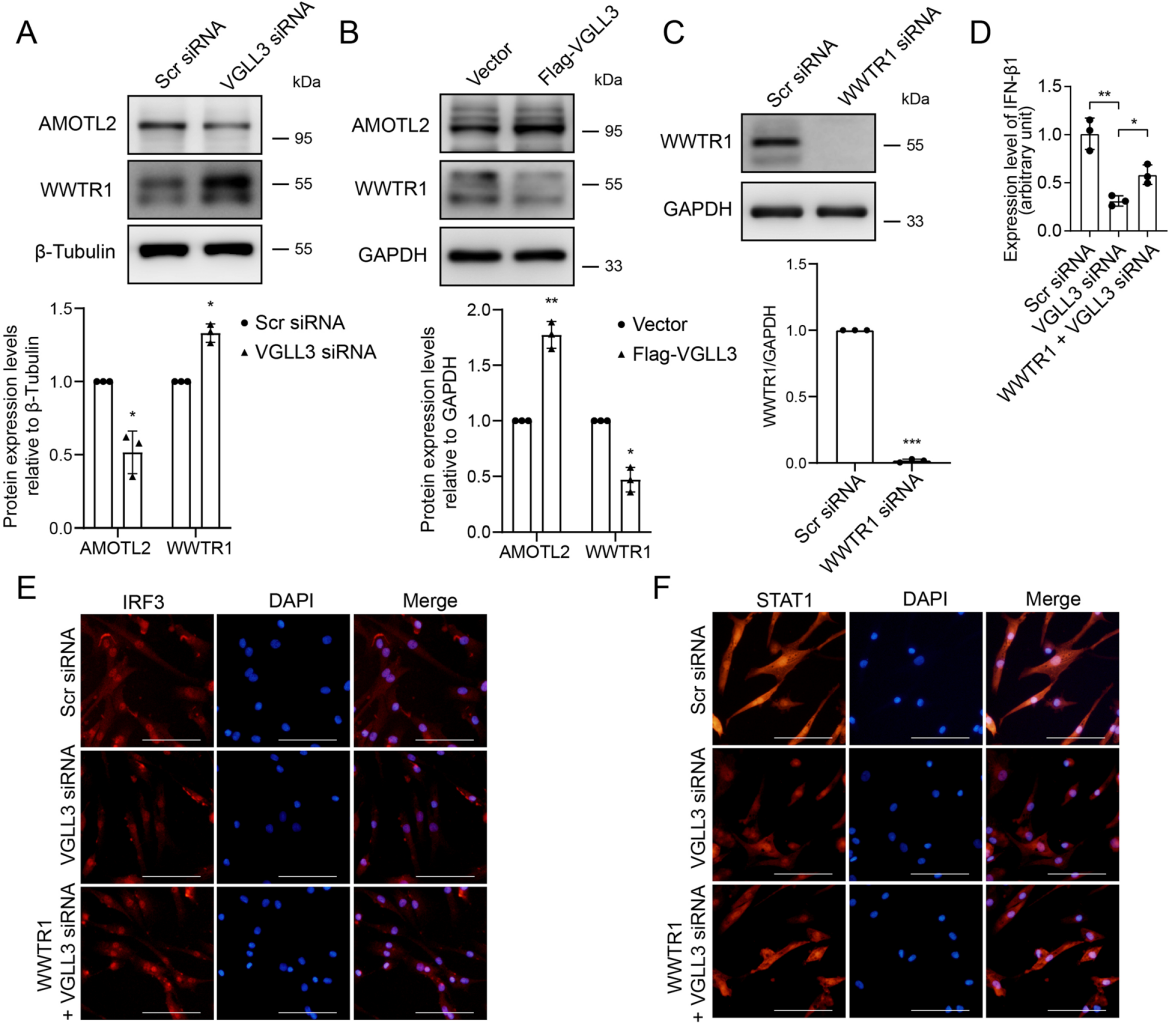
VGLL3 exacerbated IFN-β1 production by inhibiting Hippo pathway. **a** Representative images of the protein expression of WWTR1 and AMOTL2 in RA-FLS transfected with VGLL3 siRNA or Scr siRNA examined by Western blotting. β-Tubulin was used as the housekeeping gene. **b** Representative images of the protein expression of WWTR1 and AMOTL2 in RA-FLS transfected with Flag-VGLL3 expressing lentivirus or vector examined by Western blotting. GAPDH was used as the housekeeping gene. **c** Representative images of the protein expression of WWTR1 in RA-FLS transfected with WWTR1 siRNA or Scr siRNA examined by Western blotting. GAPDH was used as the housekeeping gene. **d**–**f** The mRNA expression levels of IFN-β1 (**d**) examined by real-time PCR and protein expression of IRF3 (**e**) and STAT1 (**f**) examined by immunofluorescence staining in RA-FLS transfected with Scr siRNA, VGLL3 siRNA, and WWTR1 siRNA + VGLL3 siRNA. *N* = 3. *p < 0.05, **p < 0.01, ***p < 0.001. Data were shown as mean ± SD

To explore the mechanism by which VGLL3 regulates the IFN-β1 production in RA-FLS, proteins involved in the Hippo pathway were examined. Western blotting showed that the protein expression level of WWTR1 was enhanced by VGLL3 knockdown, and the expression of AMOTL2 was inhibited (Fig. 5A). In contrast, overexpression of VGLL3 markedly suppressed the expression of WWTR1 and increased the expression of AMOTL2 (Fig. 5B). Transfection of WWTR1 siRNA into RA-FLS silenced the expression of WWTR1, as verified by Western blotting (Fig. 5C). The reduction of IFN-β1 mRNA expression level induced by VGLL3 silencing was partially rescued by WWTR1 siRNA transfection (Fig. 5D). Furthermore, WWTR1 siRNA transfection could also restore the nuclear translocation of IRF3 and the expression of STAT1 which were inhibited by VGLL3 silencing in RA-FLS (Fig. 5E, F), suggesting that VGLL3 regulates the IRF3 nuclear translocation and IFN-β1 expression through the Hippo pathway.

## Discussion

Recently, the role of VGLL3 in autoimmune diseases has drawn much attention. VGLL3-regulated gene network has been demonstrated as a promoter of sex-biased autoimmune diseases, including lupus, systemic sclerosis, and Sjögren’s syndrome. Elevated expression of VGLL3 was discovered in normal female skin and keratinocytes compared to males and also in the skin of SLE patients compared to healthy controls [28]. VGLL3-overexpressing mice had higher rates of developing lupus-like cutaneous phenotype [33]. As is well known that RA is a sex-biased autoimmune disease. The estimated ratio of the prevalence in females against males ranged from 3:1 to 4:1 in different countries [34–36]. Our data demonstrated that the expression of VGLL3 was higher in the RA synovium and RA-FLS compared to OA.

In human keratinocytes and monocytes, VGLL3 was required for the optimal expression of ITGAM and TNFSF13B, which are pro-inflammatory genes related to SLE [28]. Meanwhile, VGLL3 overexpression in mice elevated the expression of cutaneous lupus-related genes, including TNFSF13B, IFN-κ, and CXCL13 [33]. Our study revealed that VGLL3 predominantly regulated ISGs (especially type I ISGs) in RA-FLS, including STAT1, TNFSF13B, MX1, OAS1, IFI6, IFIT1, ISG15, LY6E, IFITM3, IRF7, and RSAD2. Genes involved in the Toll-like receptor signaling pathway such as TLR3 and MYD88, and chemokines such as CCL5 was also regulated by VGLL3. STAT1 is one of the master signal transducers in the process of cellular responses to IFNs. Upon stimulation of IFNs, the cellular expression of STAT1 is also increased because the upstream regulatory region of STAT1 contains IFN-stimulated response elements and GAS sites, making STAT1 an ISG [37]. STAT1 was implicated in the inflammatory phenotype in RA-FLS and mouse embryonic fibroblasts [38]. There was evidence that TNFSF13B was involved in RA pathogenesis. The protein levels of TNFSF13B were increased in the serum and synovial fluid of RA patients [39]. It increased the expression of pro-inflammatory cytokines such as IL-1 and IL-6 and enhanced the differentiation of T cells into TH17 cells [10]. Bioinformatics analysis indicated that RSAD2, OAS2, MX1, and ISG15 might be remarkable gene signatures in RA development by regulating immune responses [40]. Our results also proved that the expression of STAT1 and MX1 in RA synovium was higher than that in OA. STAT1 and MX1 expression levels correlated positively with VGLL3 in the RA synovium, respectively. Thus, VGLL3 might be implicated in RA pathogenesis through VGLL3-regulated genes.

To explain how VGLL3 regulated the expression of ISGs, the expression of type I IFNs was detected in RA-FLS. It has been testified that human fibroblasts produce IFN-β upon stimulation [41]. Intriguingly, our data revealed that IFN-β1 expression was strikingly regulated upon VGLL3 overexpression or knockdown in RA-FLS. This differed from the previous studies where VGLL3 overexpression failed to disturb the IFN-β expression in mouse skin [33], indicating that the VGLL3-regulated gene spectrum might vary in different cellular or disease context. The transcription factor IRF3 plays a pivotal role in the expression of IFN-β. Upon activation, IRF3 gets phosphorylated, moves into the nucleus, and transactivates the transcription of IFN-β by binding to its promoter region [42–44]. We found that VGLL3 overexpression triggered the phosphorylation and nuclear translocation of IRF3. JAK1 is the intracellular adaptor and signal transducer of IFNAR [5]. Tofacitinib selectively inhibits JAK1 and JAK3 [45], thus blocking the signals of type I IFNs binding to IFNAR. Importantly, our results suggested that blocking type I IFN signaling with Tofacitinib strongly repressed the VGLL3-induced ISGs expressions, indicating that VGLL3 might drive the IFN-β1 expression by activating IRF3 and secreted IFN-β1 stimulated the ISGs expression in RA-FLS in an autocrine manner.

It has been reported that YAP/WWTR1, which is the core protein of the Hippo pathway, antagonized the IFN-β expression by inhibiting the activation of IRF3 [46]. VGLL3 could activate the Hippo pathway and promote cell proliferation in A549 cells and MDA-MB-231 cells [27]. In our study, we found that VGLL3 knockdown could elevate the expression of WWTR1 in RA-FLS but had no influence on the expression of YAP (data not shown). In contrast, VGLL3 overexpression inhibited the expression of WWTR1 as expected. The angiomotin (AMOT) family could inhibit the nuclear translocation of YAP/WWTR1, acting as a novel Hippo pathway component [47]. Our results showed that the potent YAP/WWTR1 inhibitor AMOTL2 was inhibited after VGLL3 knockdown. Silencing WWTR1 with siRNA blocked the inhibitory effect of VGLL3 knockdown on IFN-β1 production in RA-FLS, suggesting that VGLL3 might regulate the IFN-β1 expression through the AMOTL2-WWTR1-IRF3 pathway.

## Conclusions

**Fig. 6:**
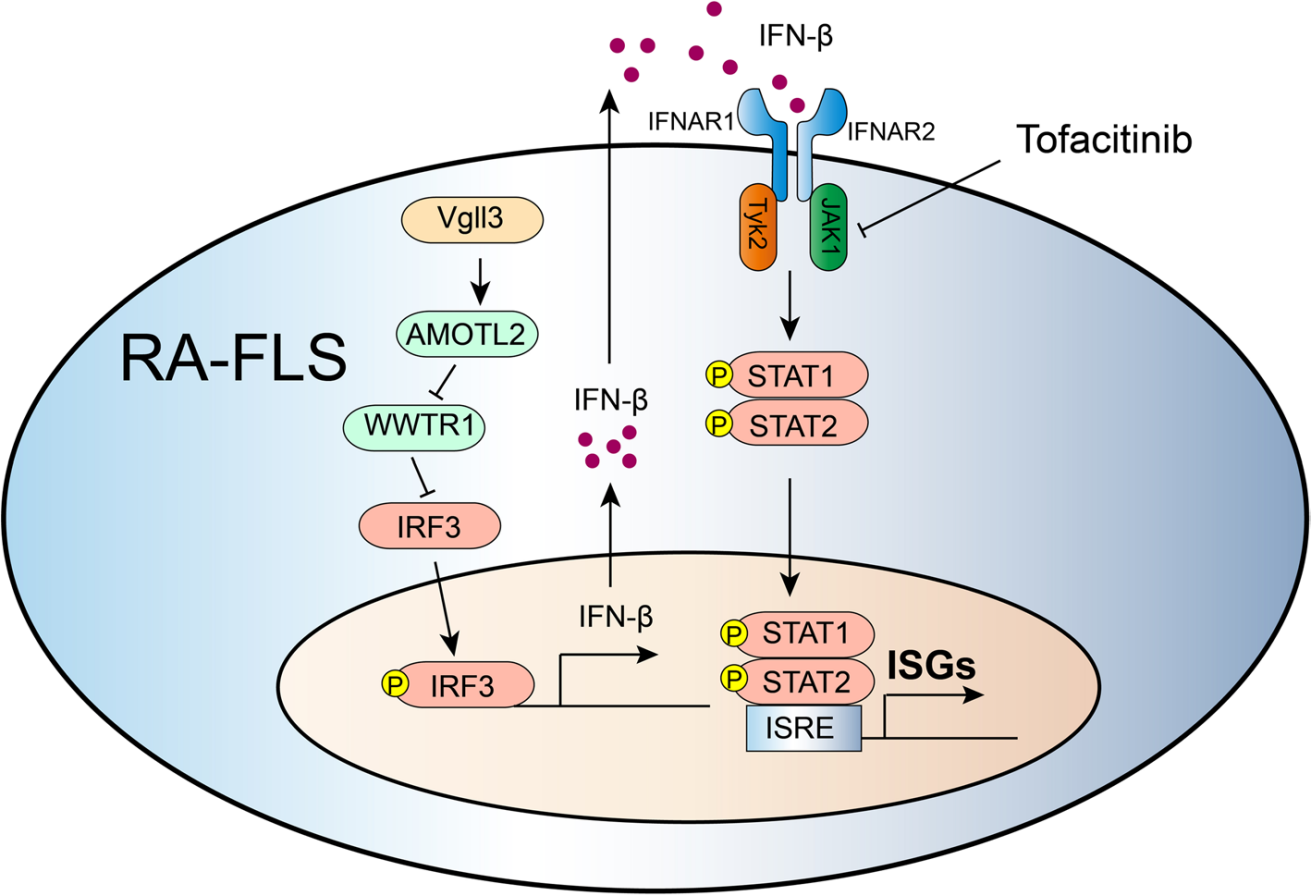
Molecular mechanism of the regulation of type I interferon signature by VGLL3 in fibroblast-like synoviocytes of rheumatoid arthritis via targeting the Hippo pathway. In fibroblast-like synoviocytes of rheumatoid arthritis, VGLL3 inhibits the expression of WWTR1 which is the core protein in the hippo pathway. By downregulating WWTR1, VGLL3 enables the phosphorylation of IRF3 and drives the IRF3-induced IFN-β1 secretion. Secreted IFN-β1 subsequently promotes the type I IFN signature expression through autocrine IFN-β1 signaling. Blocking type I IFN signaling with Tofacitinib strongly represses the VGLL3-induced ISGs expressions

VGLL3 drives the IRF3-induced IFN-β1 secretion in RA-FLS by inhibiting WWTR1 expression and subsequently promotes the type I IFN signature expression through autocrine IFN-β1 signaling (Fig. 6). These results provide novel insights into how the type I IFN signature are regulated in RA-FLS and make VGLL3 a possible RA therapeutic target.

## Data Availability

The raw data and FPKM data of RNA-sequencing generated during the current study are available in NIH Gene Expression Omnibus (GEO; accession number GSE200030). All other data generated during the current study are available from the corresponding author upon reasonable request.

## Abbreviations

AMOT: Angiomotin
ANOVA: Analysis of variance
BAFF: B cell-activating factor
BSA: Bovine serum albumin
DAB: Diaminobenzidine
DAPI: 4’,6-Diamidino-2-phenylindole
DEGs: Differentially expressed genes
DMEM: Dulbecco’s Modified Eagle Medium
FLS: Fibroblast-like synoviocytes
FPKM: Fragments per kilobase per million mapped reads
GSEA: Gene set enrichment analysis
HE: Hematoxylin and eosin
IFN: Interferon
IFNAR: IFN-α receptor
IRF9: IFN regulatory factor 9
ISGF3: IFN-stimulated gene factor 3
ISGs: IFN-stimulated genes
JAK: Janus kinase
LATS1/2: Large tumor suppressor kinase 1/2
log2(FC): log2 fold change
OA: Osteoarthritis
PFA: Paraformaldehyde
PVDF: Polyvinylidene fluoride
qPCR: Quantitative PCR
RA: Rheumatoid arthritis
RT-PCR: Reverse transcription PCR
SDS–PAGE: Sodium dodecyl sulfate-polyacrylamide gel electrophoresis
SLE: Systemic lupus erythematosus
STAT1: Signal transducer and activator of transcription 1
TAZ: Transcriptional coactivator with PDZ-binding motif
TEADs: TEA domain-containing transcription factors
TNFSF13B: Tumor necrosis factor ligand superfamily member 13B
VGLL3: Vestigial family member 3
WWTR1: WW domain-containing transcription regulator 1
YAP: Yes-associated protein
